# Is there evidence for neurodegenerative change following traumatic brain injury in children and youth? A scoping review

**DOI:** 10.3389/fnhum.2014.00139

**Published:** 2014-03-19

**Authors:** Michelle L. Keightley, Katia J. Sinopoli, Karen D. Davis, David J. Mikulis, Richard Wennberg, Maria C. Tartaglia, Jen-Kai Chen, Charles H. Tator

**Affiliations:** ^1^Bloorview Research Institute, Holland Bloorview Kids Rehabilitation HospitalToronto, ON, Canada; ^2^Department of Occupational Science and Occupational Therapy, University of TorontoToronto, ON, Canada; ^3^Graduate Department of Rehabilitation Science, University of TorontoON, Canada; ^4^Department of Psychology, University of TorontoON, Canada; ^5^Cognitive Neurorehabilitation Sciences, Toronto Rehabilitation InstituteToronto, ON, Canada; ^6^Department of Psychology and Division of Neurology, Sickids Hospital for Sick ChildrenToronto, ON, Canada; ^7^Division of Brain, Imaging and Behaviour – Systems Neuroscience, Toronto Western Research Institute, University Health NetworkToronto, ON, Canada; ^8^Department of Surgery and Institute of Medical Science, University of TorontoToronto, ON, Canada; ^9^Krembil Neuroscience Centre, Toronto Western Hospital, University Health Network and University of TorontoToronto, ON, Canada; ^10^Neuropsychology/Cognitive Neuroscience Unit, Montreal Neurological InstituteMontreal, QC, Canada

**Keywords:** traumatic brain injury, neurodegeneration, magnetic resonance imaging

## Abstract

While generalized cerebral atrophy and neurodegenerative change following traumatic brain injury (TBI) is well recognized in adults, it remains comparatively understudied in the pediatric population, suggesting that research should address the potential for neurodegenerative change in children and youth following TBI. This focused review examines original research findings documenting evidence for neurodegenerative change following TBI of all severities in children and youth. Our relevant inclusion and exclusion criteria identified a total of 16 articles for review. Taken together, the studies reviewed suggest there is evidence for long-term neurodegenerative change following TBI in children and youth. In particular both cross-sectional and longitudinal studies revealed volume loss in selected brain regions including the hippocampus, amygdala, globus pallidus, thalamus, periventricular white matter, cerebellum, and brain stem as well as overall decreased whole brain volume and increased CSF and ventricular space. Diffusion Tensor Imaging (DTI) studies also report evidence for decreased cellular integrity, particularly in the corpus callosum. Sensitivity of the hippocampus and deep limbic structures in pediatric populations are similar to findings in the adult literature and we consider the data supporting these changes as well as the need to investigate the possibility of neurodegenerative onset in childhood associated with mild traumatic brain injury (mTBI).

## Introduction

One of the most commonly reported injuries in children who participate in sports is concussion or mild traumatic brain injury (mTBI) (Browne and Lam, 2006). Children and youth (<19 years) involved in organized contact sports are nearly six times more likely to suffer a severe concussion compared to other leisure physical activities (Browne and Lam, 2006). The recovery profile and breadth of consequences in children and youth remains largely unknown (McCrory et al., 2004). This dearth of literature is compounded by the recent scrutiny youth participation in competitive contact sports (such as boxing, hockey and football) has received, due primarily to case study and media reports linking repeat concussions to a distinct neurodegenerative disease known as chronic traumatic encephalopathy (CTE).

This condition was initially described in boxers in 1928 by Martland and known as dementia pugilistica (Martland, 1928). In some cases, a constellation of symptoms typical of neurodegenerative disease were observed in a syndrome, and Miller coined the term “CTE” (Miller, 1966). It is now recognized in many sports in which there are repetitive concussions. CTE is defined as a slowly progressive neurodegenerative disorder associated with repeated brain trauma that manifests years after implicated concussive events (McKee et al., 2009). CTE is a neurodegenerative disease with a distinct distribution of atrophy along the amygdalo-hippocampal-septo-hypothalamic-mesencephalic continuum (McKee et al., 2009). CTE shows some similarity to the chronic effects of moderate and severe traumatic brain injury (TBI). There is demonstrated evidence for neurodegeneration in the chronic phase of moderate to severe TBI, ensuing months to years after brain injury with sub-acute atrophy within the limbic system hippocampi (Ng et al., 2008) and elsewhere (Greenberg et al., 2008; Farbota et al., 2012; Green et al., 2014). The corpus callosum (unmyelinated axons in particular) is vulnerable to the deposition of protein post-TBI, suggesting commonality with CTE (Reeves et al., 2007). Thus, generalized cerebral atrophy is a well-established consequence of moderate-to-severe TBI in adults that can be quantified from MRI studies that assess total brain volume (e.g., Bigler, 2010).

To the best of our knowledge, there are currently no scientific studies published of CTE following repetitive concussions/mTBI in children. The popularity of competitive sports coupled with the dearth of literature investigating long-term outcomes following mTBI in the pediatric population, suggests that research addressing the potential for CTE in youth following multiple mTBIs should be a public priority. This mini-review examines the available evidence on atrophy and neurodegenerative change in children and adolescents (<19 years) in the chronic stages of mild, moderate and severe TBI compared to typically developing youths. Research findings describe widespread volume reductions (e.g., Levin et al., 2000; Verger et al., 2001; Serra-Grabulosa et al., 2005; Tartaglia et al., 2005; Wilde et al., 2005, 2006, 2007, 2010, 2012; Braga et al., 2007; Spanos et al., 2007; Yuan et al., 2007; Fearing et al., 2008; Bigler, 2010; Beauchamp et al., 2011a,b) and clearly indicate that childhood TBI disrupts normal age-related neuronal processes that may persist across the life-span (see Bigler, 2013).

## Methods

### Identifying relevant studies

We chose a scoping review methodology (Mays et al., 2001) and entered the keywords mild, traumatic, brain, injury, MRI, child, chronic, long-term, and concussion combined with the Boolean operators AND and OR into PubMed, Ovid, PsychInfo, and Medline as the search engines. We also hand searched each reference list and included only published articles from January 1, 2000 to May 2, 2012 that contained human participants and were published in English. The start date of 2000 was chosen as studies published following this date contained imaging technology and methods sufficiently advanced in terms of sensitivity to detect more subtle structural changes. Foreign language material was excluded because of the cost and time in translating material. We adopted these methods for practical reasons and acknowledge that key articles may have been missed.

The various mechanisms for searching in our scoping study generated a total of 16 publications. No additional publications were identified as the study progressed. All publications were originally identified on the PubMed electronic databases and confirmed by subsequent databases searched.

### Study selection

Our initial examination of the studies indicated that our search strategy had identified a large number of irrelevant studies. Criteria to eliminate studies that did not address our central research question were developed *post-hoc* in three stages, based on increasing familiarity with the literature (Arksey and O'Malley, 2005). In Stage One we included original research articles and case studies that examined structural changes using MRI following mild, moderate and severe TBI. We included imaging studies examining adults only if the methods informed imaging techniques that could be applied to pediatric cases. We excluded meta-analyses and review articles as well as non-TBI forms of brain injury. These criteria identified 201 articles for review. During Stage Two, we narrowed our inclusion criteria to a TBI sustained during childhood and youth (defined as under the age of 19) and excluded metabolic studies which identified 71 articles. Finally during Stage Three we included only those studies that were cross-sectional or longitudinal in design and reported on neuroimaging findings of neurological degeneration obtained at least 1 year post-injury in order to identify those studies focused on the chronic effects following TBI for all subjects examined. We also excluded studies focusing solely on intentional brain trauma (i.e., inflicted abuse) to try and keep injury mechanism more similar to the biomechanical forces observed in concussion/mTBI. These criteria resulted in 16 articles for review.

Two reviewers (first and second authors) applied the inclusion and exclusion criteria to all the citations and copies of the full articles were obtained for those studies felt to “best fit” the research question. Having read the articles in full, all 16 articles were selected for inclusion in the review.

### Charting the data

We charted key items of information obtained from the primary research reports being reviewed (Arksey and O'Malley, 2005). We recorded information as follows:

- Author(s), year of publication and study location
- Study population (Brain Injury (TBI) Severity, Time Since Injury, and Mechanism)
- Study Population (Demographic Characteristics)
- Aims
- Study Design
- Structural Feature Assessed
- Behavioral Outcome Measure(s)
- Neurodegenerative Findings

## Results

### Numerical analysis of the extent, nature, and distribution of studies

#### Study design

Supplementary Table 1 summarizes the data obtained from each study. With respect to study design, 14 of the 16 studies reviewed utilized a cross-sectional design, of which 10 included a comparison group. Of the 10 studies containing a control group, seven studies individually matched participants across a number of demographic variables including age, sex, education (maternal or child), and socioeconomic status. One study matched on age and sex combined, with one study matching on age alone. Two studies described a control group consisting of children and youth of similar age who had sustained orthopedic injuries. All cross sectional studies included participants who were under the age of 19 years at the time of scanning. Two of the sixteen studies were prospective longitudinal investigations of the same cohort of children re-imaged at two time-points. One study re-imaged at 3 and 18 months post-injury while the second re-imaged at 3 and 36 months post-injury. Of these two longitudinal studies only one included a control group comprised of children of similar age who had sustained an orthopedic injury.

**Figure 1:**
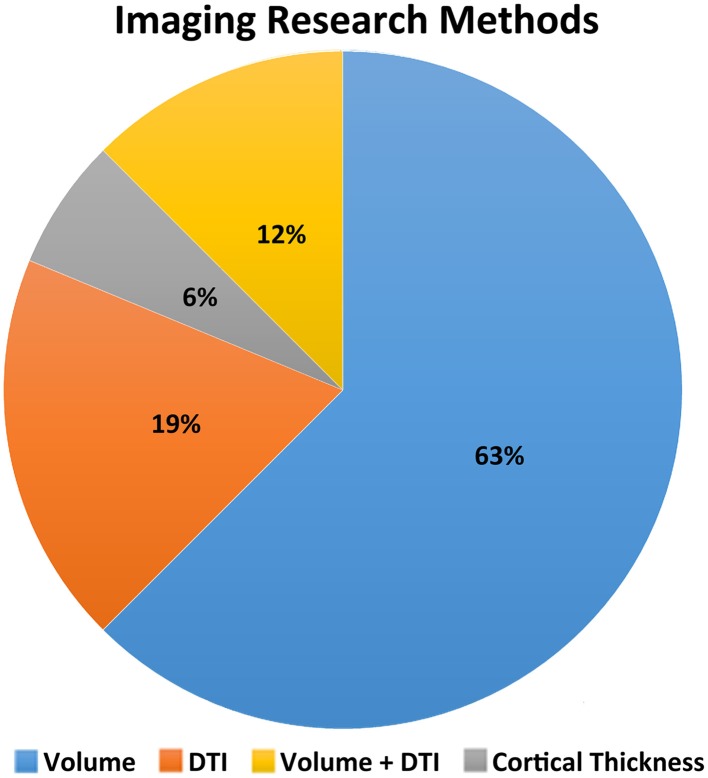
**Illustrative summary of imaging research methods**.

Of the 16 studies reviewed, eight examined volumetric properties of selected brain regions only, while three considered multiple brain regions and/or all gray and white matter (see Figure 1). Two studies report on diffusion tensor imaging (DTI) in selected brain regions (corpus callosum Wilde et al., 2006 and cingulum bundle Wilde et al., 2010, respectively), while one study used DTI to examine selected white matter regions including the corpus callosum, interior capsule, superior longitudinal fasciculus and inferior fronto-occipital fasciculus (Yuan et al., 2007). Two studies employed both volumetric and DTI methods to explore evidence for impaired brain growth across the whole brain following TBI.

#### Patient population

Of the sixteen studies reviewed, five present various findings from a single cohort of sixteen children and youth who previously sustained a moderate-severe TBI. Six studies in total considered the moderate to severe patient population. Three studies included mild, moderate and severe case while two included “complicated mTBI” [defined as children exhibiting focal pathology on acute computed tomography (CT), regardless of having GCS scores in the range of 13–15] in addition to children and youth survivors of moderate and severe TBI. One study considered the full spectrum of TBI including mild, moderate and severe TBI. One study defined their patient population as mild to moderate and severe, while a final three studies considered severe TBI only.

#### Behavioral outcome measures

**Figure 2:**
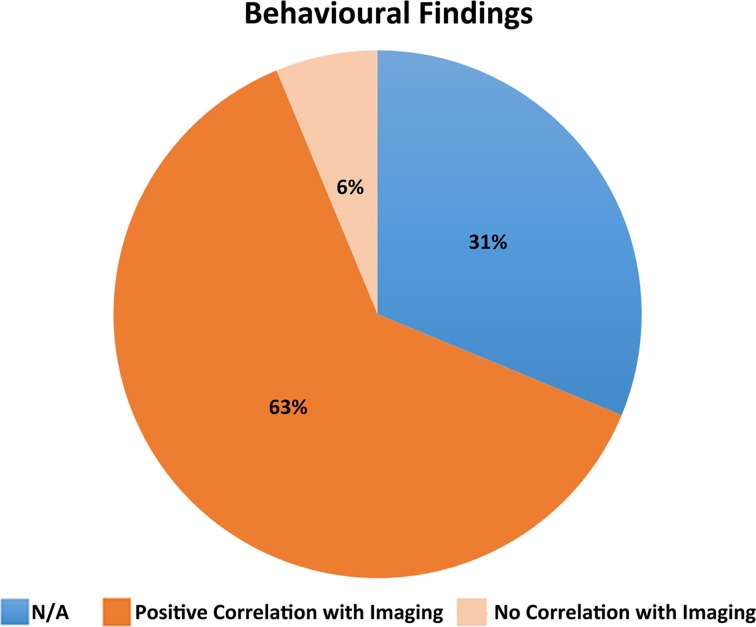
**Illustrative summary of behavioral findings**.

Just over two thirds (11/16) of the studies correlated a measure of behavioral function with the MRI findings. A total of five studies did not include a behavioral outcome measure and three studies (Wilde et al., 2005, 2006; Yuan et al., 2007) correlated MRI findings with GCS or Glasgow Outcome Scale (GOS) alone (see Supplementary Table 1). Most studies that included a behavioral outcome measure reported positive correlations with the structural features assessed (see Figure 2). For example, Wilde et al. (2012) found a significant positive correlation between the emotional control subscale of the Behavior Rating Inventory of Executive Function (BRIEF) and right medial frontal and right anterior cingulate gyrus volume. Levin et al. (2000) reported that the uncorrected corpus callosum area was correlated with acute TBI severity and Vineland Adaptive Behavior Scale (VABS) score at 36 months postinjury. Wilde et al. (2010) reported a significant correlation between a low GCS score and high apparent diffusion coefficient (ADC). Furthermore, for the TBI group, significant correlations were found between DTI parameters and behavioral measures. Fearing et al. (2008) found a decreased baseline RT on the Sternberg task to be associated with total brainstem volume for both the control and TBI groups. Yuan et al. (2007) found GCS scores to be positively correlated with FA in several white matter areas including the inferior fronto-occipital fasciculus. Braga et al. (2007) observed lesion volume and presence of lesions left supramarginal gyrus in splenium to be significantly associated with dyscalculia. Wilde et al. (2006) found higher FA was related to increased cognitive processing speed and faster interference resolution. In the TBI patients, higher FA was also related to better functional outcome as measured by the GOS. Serra-Grabulosa et al. (2005) reported verbal long term memory to be significantly correlated with volume of cerebrospinal fluid (CSF) in the TBI group only. Hippocampal volume also correlated with visual and verbal long term recall for TBI subjects. Wilde et al. (2005) observed that greater tissue preservation predicted better recovery on the GOS. Finally, Verger et al. (2001) found that corpus callosum area strongly correlated with several measures involving processing speed and visuospatial function.

### Chronic atrophy and neurodegenerative findings

#### Longitudinal studies

***Cortical thickness and volumetric changes.*** Longitudinal investigation of cortical thickness revealed that at 18 months (relative to 3 months) post-injury, bilateral frontal, fusiform, and lingual regions remained significantly decreased in TBI relative to orthopedic controls with additional areas of cortical thinning emerging in bilateral frontal regions, fusiform gyrus and left parietal regions. Wilde et al. (2012) found large bilateral regions of the medial aspects of the frontal lobes and anterior cingulate were attenuated. Most notably, there were also cortical thickness increases in aspects of the medial orbital frontal lobes and bilateral cingulate and right lateral orbital frontal lobe (Wilde et al., 2012) which could be interpreted as either compensatory hypertrophy or random effects. In addition, Levin et al. (2000) report that corpus callosum area decreased from 3 to 36 months in severely injured children and increased in the mild to moderate group. Uncorrected corpus callosum area was correlated with acute TBI severity and functional outcome at 36 months post-injury.

***White matter integrity assessed by diffusion tensor imaging (DTI).*** None of the longitudinal studies examined this parameter.

#### Cross-sectional studies

***Cortical thickness and volumetric changes.*** All eleven studies focusing on volumetric changes reported positive findings indicative of long-term degeneration in selected brain regions. More specifically, relative to a control group with similar demographic characteristics and in some cases, an orthopedic or extracranial injury, volume loss was evident in the hippocampus, amygdala, globus pallidus, thalamus (gray matter only), periventricular white matter, cerebellum, and the midbrain of the brainstem. Whole brain volume was found to be significantly decreased in TBI patients relative to controls while CSF and ventricular space was observed to be significantly greater. A number of studies attempted to control for the presence of focal lesions by analyzing volumes in brain regions with no focal lesions, as well as patients who did not have focal injuries. These, studies reported reduced volumes in selected brain regions suggesting that the degeneration was not secondary to acute injury and resulting atrophy. Finally in the one study that included mTBI and stratified results according to severity (Beauchamp et al., 2011a,b), significantly reduced gray matter and left hippocampal volume was reported for mild injuries as well as significantly increased CSF compared to an age and sex matched sample.

***White matter integrity assessed by diffusion tensor imaging (DTI).*** All five studies employing DTI reported positive findings indicating compromised white matter integrity at least 1 year following TBI compared to demographically similar control subjects, some of whom sustained an orthopedic or extracranial injury. More specifically, fractional anisotropic (FA) values were significantly reduced in the genu, body and splenium of the corpus callosum, anterior limb of the posterior capsule, posterior limb of the anterior capsule, superior fronto-occipital fasciculus, superior longitudinal fasciculus, superior fronto-occipital fasciculus, and centrum semiovale. Moreover, FA values were significantly reduced bilaterally in the cingulum bundles, while ADC values were significantly increased. Similarly, TBI patients demonstrated significantly higher mean diffusivity in the right cerebral white matter, bilaterally in the forceps major and in the body and splenium of the corpus callosum.

## Discussion

Taken together, the studies reviewed suggest there is evidence for long-term neurodegenerative change following TBI in children and youth. In particular both cross-sectional and longitudinal studies revealed volume loss in selected brain regions including the hippocampus, amygdale, globus pallidus, thalamus, periventricular white matter, cerebellum, and brain stem as well as overall decreased whole brain volume and increased CSF and ventricular space. DTI studies also report evidence for decreased axonal integrity, particularly in the corpus callosum (Wilde et al., 2006; Yuan et al., 2007; Porto et al., 2011). Although fewer in number, longitudinal investigations are of critical importance and those reviewed here (i.e., Wilde et al., 2012) highlight the dynamic and disruptive interplay between childhood TBI and normal developmental neuronal processes such as axonal thinning and increased myelination (see Bigler, 2013).

Taken together, the findings appear to highlight a sensitivity of the hippocampus and deep limbic structures in pediatric populations, which like adults, show similarities to CTE where there is a distinct distribution of atrophy along the amygdalo-hippocampal-septo-hypothalamic-mesencephalic continuum (McKee et al., 2009). They also corroborate findings in the chronic phase of moderate to severe TBI in adults, where sub-acute atrophy within the limbic system hippocampi (Ng et al., 2008), corpus callosum (Reeves et al., 2007), and elsewhere (i.e., Greenberg et al., 2008; Bigler, 2010; Green et al., 2014) have been documented.

A major limitation of the studies reviewed is the lack of studies focused specifically on repetitive concussions or mTBIs. Only one study (Beauchamp et al., 2011a,b) reported results specific to mTBI where reduced gray matter and left hippocampal volume was reported for mild injuries as well as significantly increased CSF compared to an age and sex matched sample. The second important limitation is that the methods of only a subset of the studies speak directly to a progressive, and putatively neurodegenerative entity. Chronic findings in the rest of the studies reviewed may alternatively reflect the enduring effects of the initial injuries. These findings indicate that long-term investigation of neurodegenerative change following repetitive concussions and mTBIs in children is warranted (Tartaglia et al., 2014).

There is widespread belief that children are at an advantage to adults when inflicted with significant brain damage, such as repeat concussions or mTBIs, as the developing brain has a higher chance of reorganization or plasticity (McCrory et al., 2004). This view is becoming increasingly challenged. The developing brain is cognitively maturing throughout childhood and any impact may cause a disruption in this neuronal maturation (Anderson et al., 2001). Although the injury may occur in the same way, the outcome needs to be treated differently as the composition and mechanical properties of the head and brain differ in an adult and youth (Kirkwood et al., 2006). These differences include increased brain water content, decreased level of myelination, skull geometry, suture elasticity, and neck strength (Bauer and Fritz, 2004; Kirkwood et al., 2006).

In conclusion, the mini-review provides strong evidence for neurodegenerative change following TBI of all severities in children and youth while clearly highlighting repetitive and chronic mTBI in children and youth as an overlooked population. Future research should employ multi-centerd strategies to longitudinally investigate the possibility of neurodegenerative onset and CTE in childhood associated with repeat mTBIs by developing age specific normal databases for each of the imaging parameters under assessment.

### Conflict of interest statement

The authors declare that the research was conducted in the absence of any commercial or financial relationships that could be construed as a potential conflict of interest.
