# Supplementary material for: Is there evidence for neurodegenerative change following traumatic brain injury in children and youth? A scoping review
Source: Front Hum Neurosci. 2014 Mar 19;8:139. doi: 10.3389/fnhum.2014.00139 (PMC3958726; doi:10.3389/fnhum.2014.00139)
Supplement: Supplementary file 1 [file DataSheet1.PDF]

| **Author, Year of Publication and Study Location** | **Study Population: Traumatic Brain Injury (TBI) Severity, Time Since Injury and Mechanism** | **Study Population: Demographics** | **Aims** | **Study Design** | **Structural Feature Assessed** | **Behavioural Outcome Measures** | **Neurodegenerative Findings** |
|---|---|---|---|---|---|---|---|
| Wilde et al. (2012) USA | Moderate to severe; Mean time since injury 3 and 18 months respectively; Mode of injury motor vehicle accident (MVA) | TBI group: n = 20; mean age at 3 months 13.6 years; mean age at 18 months 14.8 years; 11 males, 9 females<br><br>Control group (orthopedic injury - OI):<br>n = 21; mean age at 3 months 12.1 years mean age at 18 months 13.2 years; 15 males, 6 females | Assess patterns of cortical development over time in children who had sustained TBI compared to children who had sustained OI | Longitudinal | Volume | Behaviour Rating Inventory of Executive Function (BRIEF) | 3 months post-TBI:<br>Decreased cortical thickness bilaterally in superior, dorsolateral and orbital frontal regions, and the anterior cingulate;<br>18 months post-TBI:<br>Bilateral frontal, fusiform and lingual regions remained significantly decreased with additional areas of cortical thinning emerging in bilateral frontal, fusiform gyrus and left parietal regions;<br>Cortical thickness increases in right lateral and medial aspects of the orbital frontal lobe and bilateral cingulate. |
| Levin et al. (2000) USA | Mild to moderate and severe; Mean time since injury approximately 3 years; Mechanism reported only as high and low velocity | TBI group:<br>n=53 (28 mild to moderate and 25 severe); mean age 10.3 years; 35 males, 18 females<br><br>Control Group:<br>None | To study effects of TBI severity on development of corpus callosum in children, using Magnetic Resonance Imaging (MRI) | Longitudinal | Volume | Vineland Adaptive Behavior Scale (VABS) | Corpus callosum area decreased from 3 to 36 months in severely injured children and increased in the mild to moderate group |

| | | | | | | | |
|---|---|---|---|---|---|---|---|
| Beauchamp et al. (2011a) Australia and Canada | Mild, moderate and severe; Mean time since injury 10.4 years; Mechanism not reported | TBI group: n = 49 (11 mild, 26 moderate, 12 severe); mean age 16.98 years; 31 males, 18 females<br><br>Control group: (comparable age and gender distribution) n= 20; mean age 15.8 years; 13 males, 7 females | Examine overall brain volumes and integrity of hippocampus and amygdala in children with TBI 10 years post-injury. | Cross-sectional | Volume | None | Comparison group had less cerebral spinal fluid, more gray matter volume overall and larger left hippocampus than all TBI groups and larger right amygdala volume than mild and moderate;<br>Severe group had less white matter volume than comparison and moderate groups, but larger right amygdala versus mild and moderate. |
| Beauchamp et al. (2011b) Australia and Canada | Mild, moderate and severe; Mean time since injury 10 years; Falls (n=27); bike/pedestrian/passenger (n=25) | TBI group: n = 52 (11 mild 28 moderate 13 severe); mean age 16.4 years; 24 males, 28 females<br><br>Control group: (age matched) n = 44; mean age of 15.8 years; 18 males, 26 females | Investigate the visual appearance of the corpus callosum 10 years after childhood TBI. | Cross-sectional | Volume | Full Scale Intelligence Quotient (FSIQ) and Processing Speed Index (PSI) of Wechsler Intelligence Scale for Children-Third Edition (WISC-3) or Wechsler Adult Intelligence Scale-Third Edition (WAIS-3); Adaptive Behaviour Assessment System – Second Edition (ABAS-2) | Compared to controls, TBI group had significantly less uniform normal corpus callosum volume and more multifocal and focal thinning in the posterior body of the corpus callosum. TBI group also had significantly more individuals with moderate thinning. |

| | | | | | | | |
|---|---|---|---|---|---|---|---|
| Porto et al. (2011) Germany | Complicated mild, moderate or severe TBI; Mean time since injury 19 years; Mechanism: primarily MVAs | TBI group: n = 12; mean age 26.5 years; all male Control group: (comparable age distribution) n = 14; mean age 26 years; all male | To detect neuromorphological changes in adult survivors of childhood TBI, who showed no detectable axonal injury on conventional MRI | Cross-sectional | Volume, mean diffusivity and fractional anisotropy (FA) | None | Volume: TBI group had significantly reduced global white and grey matter volumes, and white matter within the callosal splenium bilaterally. Mean Diffusivity and Fractional Ansiotropy: Patients had greater mean diffusivity (MD) on diffusion tensor imaging (DTI) in right posterior periventricular cerebral white matter, and suggestive areas of increased MD bilaterally in forcepts major, splenuim and body of corpus callosum. Trend of decreased fractional anisotropy (FA) in right cerebral white matter in superior parietal lobule and posterior part of corona radiata on the path of the superior longitudinal fascicle. |
| Wilde et al. (2010) USA | Complicated mild to moderate and severe; Mean time since injury 4.1 years; Primarily MVAs | TBI group: n=46 (29 severe, 8 moderate, 9 complicated mild); mean age 13.5 years; 32 males, 14 females Control group: (orthopedic injury) n=43; mean age 12.1 years; 31 males, 12 females | Determine if TBI affected the structural integrity of the cingulum bundle, and to examine if structural injury is related to cognitive control | Cross-sectional | Fractional anisotropy (FA) and apparent diffusion coefficient (ADC) | Eriksen Flanker Task, Sternberg Item Recognition Task | Higher FA emanating from bilateral cingulum bundle of OI group. Higher apparent diffusion coefficient in TBI. Higher ADC in TBI injured as a result of high-speed vs. low-speed mechanism. |

| | | | | | | | |
|---|---|---|---|---|---|---|---|
| Fearing et al. (2008) USA | Moderate to severe; Mean time since injury 3.1 years; Primarily MVAs | TBI group: n=16 mean age, 12.9 years; 8 males, 8 females Control group: (age and gender matched) n=16 years, mean age 12.8 years; 8 males, 8 females | Examine changes in morphometry of the thalamus and brainstem and correlate volumetric changes in these structures with measures of cognitive functioning | Cross-sectional | Volume | Sternberg item recognition task (memory load on processing time) | TBI group showed thalamic grey matter reduction, total midbrain volume reduction (including tectum and tegmentum volumes individually). TBI group also showed trend for reduced brainstem volume. |
| Yuan et al. (2007) USA | Mild, moderate and severe (2 severe, 7 moderate with 4 of them mild with abnormal imaging); Minimum 1 year after injury (mean time since injury not reported); Mechanism not reported | TBI group: n=9, mean age 7.89, 6 males, 3 females Control group: (orthopedic injury) n=12; mean age 7.51 years; 7 males, 5 females | To examine alteration of fractional anisotropy (FA) in children with TBI experienced during early childhood and to quantify the association between FA and injury severity | Cross-sectional | Fractional ansiotropy (FA) | Glasgow Coma Scale (GCS) | FA values were significantly reduced in the TBI group in the genu of corpus callosum, posterior limb of internal capsule, superior longitudinal fasciculus, superior fronto-occipital fasciculus, and centrum semiovale. |

| | | | | | | | |
|---|---|---|---|---|---|---|---|
| Spanos et al. (2007) USA | Moderate to severe; Mean time since injury 3 years; Vehicle, bicycle or pedestrian related motor vehicle crashes | TBI group: n=16; mean age 13 years, 8 males, 8 females Control group: (matched for age, sex, handedness, ethnicity and maternal education) n=16, mean age 13 years; 8 females and 8 males | Morphometric analysis of cerebellar grey and white matter changes in children with TBI. Explored the contribution of focal-versus-diffuse injury processes; Examined the relation between cerebellar volume and the volume of known sites of its projection | Cross-sectional | Volume | None | TBI had reduced white matter volume in the entire cerebellum. A positive relationship between white matter volume in dorsolateral prefrontal cortex and cerebellum in controls but not in the TBI group. |
| Braga et al. (2007) Brazil | Severe; Mean time since injury 4.5 years; Mechanism not reported | TBI group: n=23; mean age 11.1 years; 13 males, 10 females Control group: None | MRI assessment of brain lesion topography and volume and to investigate their relationships with neuropsychological outcomes in children and adolescents who sustained a severe TBI with focus on role of deep (especially cerebellar) brain lesions | Cross-sectional | Volume | WISC, structured parent interview, and Physical–Functional Classification of the Child and Adolescent | The most frequently affected brain areas were the corpus callosum (74%) and the left and right frontal lobes (61% and 52%, respectively). Overall, sixty-one percent (61%) of the children had cerebral atrophy. |

| | | | | | | | |
|---|---|---|---|---|---|---|---|
| Wilde et al. (2007)<br>USA | Moderate to severe;<br>Mean time since injury 3.1 years;<br>Primarily MVAs | <u>TBI group</u>:<br>n=16 mean age, 12.9 years; 8 males, 8 females<br><u>Control group</u>:<br>(age and gender matched)<br>n=16 years, mean age 12.8 years; 8 males, 8 females | To compare volumetric measurements of the hippocampus, amygdala, globus pallidus, putamen, and caudate in TBI versus controls, compare the relative volume loss in these structures with volume loss in cortical areas, and examine whether volumetric changes in these structures are related to focal injury as opposed to diffuse or secondary injury | Cross-sectional | Volume | None | Smaller volumes in total hippocampal, amygdala, and globus pallidus volumes in TBI versus control group. Marginally disproportionate volume loss in the hippocampus versus the rest of the temporal lobes, and a significant relative vulnerability when compared to the amygdala. |
| Wilde et al. (2006)<br>USA | Moderate to severe;<br>Mean time since injury 3.1 years;<br>Primarily MVAs | <u>TBI group</u>:<br>n=16 mean age, 12.9 years; 8 males, 8 females<br><u>Control group</u>:<br>(age and gender matched)<br>n=16 years, mean age 12.8 years; 8 males, 8 females | Measured anterior commissure cross-sectional volume, and explore relationship between anterior commissure and temporal stem using DTI in temporal lobe-anterior commissure fiber stem | Cross-sectional | Fractional anisotropy (FA) | Glasgow Outcome Score (GOS) and Eriksen Flanker Task | FA was significantly lower in the patients for the genu, body, and splenium of the corpus callosum.<br>FA also increased as a function of the area of specific regions of the corpus callosum such as the genu and splenium, and FA in the splenium was reduced with greater volume of lesions in this region. |

| | | | | | | | |
|---|---|---|---|---|---|---|---|
| Tasker et al. (2005)<br>UK | Severe;<br>Mean time since injury 5.8 years;<br>Mechanism not reported | <u>Non-intracranial pressure (ICP) group</u>:<br>n=33; mean age and sex distribution for not reported<br><u>ICP group</u>:<br>n=23; mean age and sex distribution not reported<br><u>Control group</u>:<br>None | The objective was to consider the interaction among severity of TBI in childhood, white matter pathology, growth anthropometry, and hippocampal atrophy | Cross-sectional | Volume and fractional anisotropy (FA) | None | Eight patients had periventricular white matter loss and smaller-than-expected brain volume for orbitofrontal cortex, suggesting atrophy; the remainder had expected volume for a smaller orbitalfrontal cortex, suggesting growth disturbance.<br>Higher eigenvalues were found in the right hemisphere for the ICP group who also had voxel-based morphometry tissue loss. |
| Serra-Grabulosa et al. (2005)<br>Spain | Severe;<br>Mean time since injury 9.7 years;<br>Mechanism not reported | <u>TBI group</u>:<br>n=16, mean age 17.9 years; 14 males, 2 females<br><u>Control group</u>:<br>(matched for age, gender, education and socioeconomic status) n=16; mean age 16.9 years; 14 males, 2 females | To investigate the cerebral correlates of memory impairment in teens who suffered severe TBI in childhood. | Cross-sectional | Volume | Rey Auditory Verbal Learning Test (RAVLT), Rey-Osterrieth Complex Figure Test (RCFT) | TBI subjects had global brain white matter volume atrophy, frontal white matter atrophy, bilateral hippocampal reduction and increase in cerebrospinal fluid volume. |

| | | | | | | | |
|---|---|---|---|---|---|---|---|
| Wide et al. (2005) USA | Moderate to severe; Mean time since injury 3.1 years; Primarily MVAs | TBI group: n=16 mean age, 12.9 years; 8 males, 8 females Control group: (age and gender matched) n=16 years, mean age 12.8 years; 8 males, 8 females | MRI volumetry was used to evaluate brain volume differences in the whole brain, and prefrontal, temporal, and posterior regions of children following moderate to severe TBI | Cross-sectional | Volume | Glasgow Outcome Scale (GCS) | TBI group had reduced whole brain, prefrontal and temporal regional tissue volumes, and increased cerebrospinal (CSF) volume and ventricle-to-brain ratio. Group differences on grey matter and white matter in the superior medial and ventromedial prefrontal cortex, white matter in the lateral frontal region, and grey matter, white matter and cerebrospinal fluid in the temporal region. Grey matter loss in the frontal areas was primarily attributable to focal injury, while white matter loss in the frontal and temporal lobes was related to diffuse and focal injury. |
| Verger et al. (2001) Spain | Moderate to severe; Mean time since injury 9.4 years; Mechanism not reported | TBI group: n=19; mean age 18.1 years; 17 males, 2 females Control group*: (matched for age, sex, education and socioeconomic status) n=19; mean age not reported; 17 males, 2 females | To investigate the relationship between MRI atrophy parameters (ventricular dilation and corpus callosum reduction) and the longterm neuropsychological sequelae detected by neuropsychological tests | Cross-sectional | Volume | WAIS, RAVLT, RCFT, Judgment of Line Orientation, Wisconsin Card Sorting Test, Controlled Oral Word Fluency, Verbal Fluency | The mean and standard deviation for corpus callosum area were 551.28 ± 111.44 $mm^2$ (range 360 - 809). The mean and standard deviation for ventricular volume were 21 208.79 ± 12 679.98 $mm^3$ (range 6746 - 45 599), and for intracranial volume estimation 777 141.28 ± 56 481.64 $mm^3$ (range 564 014.36 - 847 496.60). |

**Table 1: Table of Evidence**

***Note**: Control group did not undergo MRI protocol thus no comparison is available with respect to structural features assessed.**
